# Comparison of slot wall deformation in conventional and self-ligating brackets during torque with TMA wire - A finite element analysis

**DOI:** 10.6026/973206300222122

**Published:** 2026-04-30

**Authors:** C Keerthana, P Siri Krishna, Pavan Kumar, Manjunath Hegde, C Chaitra

**Affiliations:** 1 Department of Orthodontics and Dentofacial Orthopedics, Bangalore Institute of Dental Sciences, Bengaluru, Karnataka, India

**Keywords:** Torque, titanium molybdenum alloy (TMA) wire, slot wall deformation, FEM, self-ligating bracket, ceramic bracket

## Abstract

Slot wall deformation occurs when twisting of the wire is increased. Therefore, it is of interest to evaluate deformation in conventional, active and passive self-ligating stainless steel and ceramic brackets with 0.019 X 0.025inch TMA wire. Deformation of slot wall with TMA wire was least in ceramic passive self- ligating bracket ranging from 0.02 to 0.50 mm and highest in Stainless steel conventional bracket anging from 0.097 to 2.04 mm. Sequence of brackets from least to high slot wall deformation is Ceramic Passive Self-ligating bracket, Ceramic Active Self-ligating bracket, Stainless steel Passive Self-ligating bracket, Ceramic conventional bracket, Stainless steel Active Self-ligating bracket and Stainless-steel conventional bracket. Thus, elastic deformation of stainless-steel bracket slot was more than ceramic bracket slot.

## Background:

Orthodontic brackets are the passive components of fixed appliance therapy which transfer the forces from the arch wire to the teeth to enable tooth movement. Final root positioning of the teeth are vital for long-term stability, function and esthetics and are usually done by torquing. Torque is expressed to bring about tooth movement and this occurs due to transfer of force from the wire to the tooth through the slot of the bracket. The variables in the expression of torque includes: Stiffness of wire alloys, Play between wire and slot, Ligation modes and Bracket design [1]. As Torqueing Forces Are exerted inside the bracket slot, it is necessary to study the deformation inside the slot-i.e. in the slot wall regions. A deformation in slot wall might lead to either temporary or permanent changes in slot dimensions, leading to changes in the torque applied to the bracket slot [2]. The archwire twist (torque) imparts significant forces inside the bracket slot. In some cases, where additional torque is required, twisting of the wire is increased which leads to slot wall deformation. Therefore, it is essential to know the bracket and wire combination that will cause least slot wall deformation. Self-ligating brackets are pre-adjusted appliances with a built-in mechanism to hold the archwire in the bracket slot. They have undergone major structural changes since the inception of the Russell attachment in 1935. Reduced chair time, better oral hygiene and claims of low frictional resistance have been reported for self-ligating brackets [3]. Archambault [4] showed that active self-ligating brackets had a better torque control than passive brackets and that the initial play of the archwire in the slot was less for active self-ligating brackets. Conventional and Self-ligating brackets are available in materials like Stainless steel and Ceramic. Ceramic brackets were introduced primarily to address the growing demand for aesthetic orthodontic options.

An FEM study [2] compared the deformation of stainless steel (SS) & ceramic bracket slot walls during torque. Results showed that the deformation of Stainless steel bracket slot wall was more than ceramic bracket slot wall. A study by Shin [5], demonstrated that the Active self-ligating brackets had more plastic deformation with visually identifiable warping in the bracket slot on torque application using stainless steel wire. Archwires commonly used in finishing stages are Stainless steel and Titanium Molybdenum Alloy (TMA) wires. TMA offers a highly desirable combination of strength and springiness (i.e., excellent resilience), as well as reasonably good formability. This makes it an excellent choice for auxiliary springs and for intermediate and finishing archwires, especially rectangular wires. Rectangular NiTi and beta-Ti wires offer advantages over stainless steel wire for the finishing phases of treatment and torque control [6]. Therefore, it is of interest to determine the slot wall deformation in conventional, active and passive self-ligating brackets-Metal and Ceramic with Titanium- molybdenum alloy (TMA) wire during torque using finite element analysis.

## Materials and Methods:

## Source of DATA:

For this study, microcomputer tomography of maxillary right central incisor metal and ceramic brackets: Conventional (0.022 X 0.028inch), Active self-ligating In-Ovation (0.022 X 0.028inch) and passive self-ligating Damon (0.022 X 0.028 inch) brackets were taken. The images were converted to STL file using mimics software. The STL file was imported to HyperMesh for refining and connecting the mesh and finite element models were generated. Wire model was created using Solid modeling software.

## Materials used in the study:

## Finite element models of brackets:

Conventional 3M® Bracket (0.022 X 0.028inch) (Figure 1 - see PDF):

1) Metal

2) Ceramic

Active Self-ligating In-Ovation®Bracket (0.022 X 0.028 inch) (Figure 2 - see PDF):

1) Metal

2) Ceramic

Passive Self-ligating DAMONTMQ Bracket (0.022X 0.028 inch) (Figure 3 - see PDF):

1) Metal

2) Ceramic

## Software used for the procedure:

[1] Conversion of Micro Computer tomography images to STL file - Mimics

[2] FE model generating software - HyperMesh

[3] FE analysis software - Ansys R18.1

[4] Wire model generating software - Solid modelling software

## Method of collection of data:

Micro Computer tomography of Metal and Ceramic Brackets - Conventional 3M® (0.022 X 0.028inch), Active Self-ligating In-Ovation® (0.022 X 0.028inch) and Passive Self-ligating DAMONTM Q (0.022 X 0.028inch) were taken. The image was converted into STL file (Stereo lithography - a popular 3D printing technology) using mimics software to generate 3D geometric model. The geometric models were converted into finite element models using Hyper Mesh software. Wire FE model was created using Solid modeling software. Material properties obtained from the literature were assigned to Bracket and Wire models (Table 1 - see PDF).

## Torque and force values [9]:

TMA 0.019 X 0.025-inch wire segment was twisted to find the initial contact between bracket slot wall and wire following which the wire was twisted from 10 to 40 degree in increments of 5 degree, The archwire contact points in the slot walls during torqueing from initial contact to 40 degree twists were identified graphically by twisting the archwire inside the bracket slot using Ansys R18.1 software (Figure 4 & 5 - see PDF).

By substituting the value of theta, Torque value was calculated since theta should be in radians.

Formulae for torque is changed to,

T=30769 X 0.01625 X (ϴ X 22/ (180 X 7))/10

T=0.8726Xϴ

All degrees of freedom in the bracket base were completely arrested. Torque and force values were applied as a couple in the bracket slot of the Finite element models of 3M(conventional), In-Ovation(Active self-ligating) and DAMON Q(Passive self- ligating) - Stainless steel and ceramic brackets. Slot wall deformations were measured in millimeters (Ansys R 18.1 software) Nodal deformations were measured at: Top, Middle & Bottom locations in the slot wall [9] (i.e., 0.71, 0.355, 0.05 mm respectively from the slot base) (Table 1-7 - see PDF).

## Results:

Finite Element Models of conventional, active and passive self-ligating stainless steel and ceramic brackets with 0.019 X 0.025inch TMA wire were generated. All degrees of freedom in the bracket base were completely arrested. Torque and force values were applied as a couple in the bracket slot of the Finite element models. Slot wall deformations were measured in millimeters using Ansys R 18.1 software. Nodal deformations were measured at Top, Middle & Bottom locations in the slot wall. The initial contact of the archwire with the slot wall occurred at a wire twist angle of 7 degree in conventional brackets, 7 degree in active brackets and 8 degree in passive brackets. The deformation of slot wall at initial contact is as shown in Table 8 (see PDF). In this study, the deformation of slot walls at angles of twist of 0.019 X 0.025inch TMA wire is considered from 10 to 40 degree with increments of 5 degree in Conventional, Active and Passive Self-ligating brackets - stainless steel and ceramic which were recorded as shown in Table 9 (see PDF). Deformation of slot wall with TMA wire was least in Ceramic passive self-ligating bracket ranging from 0.023 to 0.50 mm and highest in Stainless steel conventional bracket ranging from 0.09 to 2.04 mm. The deformation was increased with increase in twist in the wire. The sequence of brackets from least to greatest slot wall deformation is: Ceramic Passive Self- ligating bracket, Ceramic Active Self-ligating bracket, Stainless steel Passive Self-ligating bracket, Ceramic conventional bracket, Stainless steel Active Self-ligating bracket and Stainless steel conventional bracket. The combination of Ceramic passive self-ligating bracket and TMA wires demonstrates minimal slot wall deformation, thereby enhancing the efficiency of torque expression (Table 10-12 - see PDF).

## Discussion:

Torque is defined as the labiolingual or buccolingual inclination of the tooth position. According to Andrews's third key of occlusion i.e. Crown inclination is generally achieved through torque. Torque is expressed to bring about tooth movement and this occurs due to transfer of force from the wire to the tooth through the slot of the bracket. Such forces tend to deform the slot wall apart from moving the tooth. Deformation in slot wall might lead to either temporary or permanent changes in slot dimensions, leading to changes in the torque applied to the bracket slot [2]. The variables in the expression of torque include Stiffness of wire alloys, Play between wire and slot, Ligation modes and Bracket design [1]. TMA wire is strong, resilient and has good formability, making it ideal for auxiliary springs and intermediate and finishing archwires, particularly rectangular ones. Rectangular NiTi and beta-Ti wires are preferred over stainless steel for finishing treatment and torque control [6]. Therefore, this study uses TMA wire instead of stainless-steel wire. The FEM has reformed biomechanical research in Orthodontics, as it represents a non- invasive, accurate method that provides quantitative and detailed data regarding orthodontic biomechanics and physiological responses. Therefore, Finite element analysis was used to evaluate the slot wall deformation in this study. Finite Element Analysis was used to evaluate Conventional, active and passive self-ligating - stainless steel and ceramic brackets slot wall deformations with angles of twist of TMA wire from 10 to 40 degree with increment of 5 degree [8, 9]. This study, demonstrated that as the angle of twist of wire is increased, the length of couple arm distance is decreased leading to higher applied torque forces. The increased torque forces resulted in an increased deformation of Conventional, active and passive self-ligating stainless steel and ceramic brackets, as shown in Table 9 (see PDF) with minimum deformation seen at 10 degree twist of wire ranging from 0.023 to 0.097 mm and highest deformation seen at 40 degree twist of wire ranging from 0.50 to 2.04mm. The deformation of the slot wall is influenced by the material properties, specifically its Young's modulus. Higher the Young's modulus indicates that the material is stiffer and more resistant to stretching, whereas a lower Young's modulus results in greater deformation. Stainless steel (SS) has a lower Young's modulus compared to ceramic, resulting in greater elastic deformation of Stainless steel brackets as opposed to ceramic brackets. Ceramic brackets are increasingly preferred due to their aesthetic appeal, higher hardness and resistance to stains [10]. An FEM study [22] compared the deformation of stainless steel & ceramic bracket slot walls during torque. Results showed that the deformation of Stainless steel bracket slot wall was more than ceramic bracket slot wall. This study also showed similar results with less deformation of slot wall in ceramic brackets than Stainless steel brackets across all torque angles i.e., 10 to 40 degree twist of wire. Least slot wall deformation was observed in ceramic passive self-ligating bracket with deformation ranging from 0.023 to 0.50 mm and highest in stainless steel conventional bracket ranging from 0.097 to 2.04mm. A recent study [4] demonstrated that active self-ligating brackets provide better torque control than passive brackets and exhibit less initial play of the archwire in the slot hence showed an advantage of active over passive self-ligating brackets. However, as the brackets were measured with only the self- ligating mechanism closed, the difference in torque expression might have been more likely due to variance in slot width than the actual effect of the clips. Moreover, recent clinical investigation suggests that play is more exaggerated in passive self-ligating brackets than in active ones; a finding, which is also supported by a recent clinical investigation [7]. However, this study results showed more slot wall deformation in active self-ligating compared to passive self-ligating brackets. The bracket with the least slot wall deformation was the passive self-ligating ceramic bracket, while the Stainless-steel conventional bracket exhibited the most deformation. As the difference in deformation between active and passive self-ligating bracket was around 0.02 to 0.1mm indicating more influence of material than bracket design in slot wall deformation [11, 12]. Slot wall deformation of passive self-ligating ceramic bracket ranging from 0.02 to 0.50mm and active self-ligating ceramic bracket ranging from 0.04 to 0.67mm as shown in Table 9 (see PDF). The bracket with least slot wall deformation with TMA wire with angle of twist ranging from 10 to 40 degree was passive self-ligating ceramic bracket with least deformation at the bottom nodal position hence efficient expression of applied torque [13, 14]. Sundar *et al.* in study concluded that in both 0.018-in and 0.022-in brackets, the behavior of the 2-bracket segment-archwire-ligature combination with varying IBD was strongly influenced by the individual factors and also in combination [15, 16]. This study provides significant information to clinicians that there is torque-relevant slot wall deformation in widely used Stainless steel and ceramic conventional, active and passive self-ligating brackets. Insight into torque- relevant bracket slot wall deformation during fixed appliance therapy is cardinal to achieve ideal and stable results.

## Limitations:

The finite element (FE) model did not incorporate tooth movement or time-dependent biological response. In clinical scenarios, as torque is applied, teeth gradually move, which dissipates the force over time. This dynamic interaction between tooth movement and applied torque could significantly affect bracket deformation. Deformation of the archwire itself during torque application was not incorporated in FE model. Archwires can undergo elastic or plastic deformation, which affects force transmission to the bracket slot.

## Conclusion:

The Elastic deformation of the stainless steel bracket slot was more than that of the ceramic bracket slot. Passive self-ligating bracket had less slot wall deformation compared to active self-ligating or conventional brackets. Passive Self-ligating Ceramic Bracket showed least slot wall deformation with TMA wire.

## References

[1] Lin J (2022). Int J Clin Pract..

[2] Badawi HM (2008). Am J Orthod Dentofacial Orthop..

[3] Major TW (2011). Am J Orthod Dentofacial Orthop..

[4] Archambault A (2010). Angle Orthod..

[5] Shin SH (2021). Materials (Basel)..

[6] Maizeray R (2021). Int Orthod..

[7] Stefanos S (2010). Am J Orthod Dentofacial Orthop..

[8] Krishnan M (2009). Am J Orthod Dentofacial Orthop..

[9] Al-Thomali Yet (2017). J Clin Exp Dent..

[10] Salehi P (2024). J Orofac Orthop..

[11] Martelli K (2019). J Clin Exp Dent..

[12] Ajami S, Boroujeni AR (2018). J Clin Exp Dent..

[13] Szczupakowski A (2016). J Orofac Orthop..

[14] Harsha SB (2024). Cureus..

[15] Sundar S (2026). Am J Orthod Dentofacial Orthop,.

[16] Sundar S (2025). Proc Inst Mech Eng H..

